# Vitamin D Status and Its Association With Sun Exposure in Patients With Type 2 Diabetes Mellitus in Southern Malaysia

**DOI:** 10.7759/cureus.79747

**Published:** 2025-02-27

**Authors:** Norizzati Amsah, Zaleha Md Isa, Norfazilah Ahmad, Zaid Kassim, Siti Nor Athirah Mohamad

**Affiliations:** 1 Family Health Research Centre, Institute for Public Health, National Institute of Health, Shah Alam, MYS; 2 Public Health Medicine, Faculty of Medicine, Universiti Kebangsaan Malaysia, Kuala Lumpur, MYS; 3 Public Health Medicine, Fakulti Perubatan Universiti Kebangsaan Malaysia, Kuala Lumpur, MYS; 4 Johor State Health, Tangkak District Health Office, Tangkak, MYS; 5 Johor State Health, Segamat District Health Office, Segamat, MYS

**Keywords:** malaysia, sunlight exposure, type 2 diabetes mellitus, vitamin d deficiency, vitamin d insufficiency

## Abstract

Introduction and aim

Vitamin D deficiency and insufficiency are widely recognized as significant concerns among individuals with type 2 diabetes mellitus (T2DM). Adequate sunlight exposure is essential for synthesizing vitamin D, yet Malaysia, despite its tropical climate, faces a high prevalence of vitamin D deficiency and insufficiency. This study aimed to determine the prevalence of vitamin D deficiency and insufficiency and their relationship with sun exposure among T2DM patients in Southern Malaysia.

Methodology

A cross-sectional study was conducted among 330 T2DM patients in Johor, a southern region of Malaysia. A blood sample of 3 mL was collected from each respondent for serum vitamin D analysis. Respondents must fast for at least 8 hours before taking the blood sample. Serum vitamin D levels were analyzed via ultra-performance liquid chromatography (UPLC) at the Universiti Kebangsaan Malaysia (UKM) Medical Molecular Biology Institute (UMBI). A validated questionnaire was used to collect data on the duration of sunlight exposure on weekdays and weekends.

Results

Multinomial logistic regression analyses were used to determine the factors associated with vitamin D deficiency and insufficiency. The mean serum vitamin D level was 49.26 (SD 15.21) nmol/L. A total of 12.1% (n=40) of the respondents were classified as vitamin D deficient and 46.4% (n=153) as vitamin D insufficient. The analysis showed that the patients with sun exposure ≤30 minutes during weekdays were significantly more likely at higher risk of vitamin D insufficiency (adjusted odds ratio {AOR}: 2.28, 95% CI: 1.09-4.88) and vitamin D deficiency (AOR: 4.12, 95% CI: 1.02-6.64). A similar trend was observed on weekends. Patients with sun exposure ≤30 minutes had higher odds of having vitamin D insufficiency (AOR: 3.82, 95% CI: 1.14-2.85) and vitamin D deficiency (AOR: 4.26, 95% CI: 1.14-2.85).

Conclusion

Vitamin D deficiency and insufficiency are common among individuals with type 2 diabetes mellitus (T2DM) in Johor, with limited sun exposure identified as a significant modifiable risk factor. Addressing this issue necessitates targeted public health initiatives to enhance awareness and promote appropriate sun exposure practices, ultimately aiming to improve vitamin D status and health outcomes in this vulnerable population.

## Introduction

Malaysia is situated in Southeast Asia and experiences a tropical climate characterized by consistently high temperatures, abundant sunlight, and high humidity. The country's temperatures remain relatively stable throughout the year, typically ranging between 21°C and 32°C, providing favorable conditions for year-round sunlight exposure [1]. Even in a tropical nation with year-round sunshine, vitamin D deficiency and insufficiency are surprisingly common in Malaysia [2]. This paradox may be explained by lifestyle factors such as limited outdoor activities, the use of sun protection, and cultural practices, which reduce effective skin exposure to sunlight, thereby limiting endogenous vitamin D synthesis [2,3]. Ultraviolet B (UVB) exposure is the primary natural source of vitamin D synthesis, but its effectiveness depends on multiple factors, including skin pigmentation, time of day, and duration of exposure. Malaysians, particularly those with darker skin tones, may require longer sun exposure for adequate vitamin D production [1,2,4].

Urbanization, increased indoor work, and sedentary behaviors contribute to insufficient sun exposure in many individuals, compounding the likelihood of vitamin D deficiency [5]. According to a meta-analysis by Saffian et al., Malaysia has a high prevalence of both vitamin D deficiency and insufficiency, with the pool prevalence of vitamin D deficiency at 21% and vitamin D insufficiency at 64%, which is exceptionally high in comparison to other regions [1]. The prevalence of vitamin D deficiency is given more towards the general population in Malaysia.

Malaysia has the fifth-highest T2DM prevalence in the Western Pacific Region [6]. The National Health and Morbidity Survey (NHMS) has reported that the prevalence of T2DM in Malaysia grew from 13.4% in 2015 to 15.6% in 2023 [7]. Vitamin D deficiency is significantly more prevalent among individuals with type 2 diabetes mellitus (T2DM), highlighting a critical health concern within this population [8]. A study in India revealed that 74.14% of T2DM patients had low serum vitamin D levels [9]. According to a study conducted in Lagos, Nigeria, the prevalence of vitamin D deficiency among T2DM patients was 63.2% and a study in Singapore showed the prevalence of vitamin D deficiency was 42% [10,11]. The existing studies focused on the general population such as adult men and women, pregnant women, postmenopausal women, adolescents, and children instead of specific populations such as T2DM patients [2]. While studies from India and Nigeria report high vitamin D deficiency in T2DM patients, data from Malaysia remain scarce. No large-scale studies have specifically examined the relationship between sun exposure and vitamin D status among Malaysian T2DM patients. Given Malaysia’s rising T2DM prevalence and potential vitamin D-related metabolic implications, investigating this association is crucial. Findings from this study could help inform sun exposure guidelines for at-risk populations, potentially influencing national health recommendations on vitamin D intake and outdoor activity. Therefore, this study aimed to determine the prevalence of vitamin D deficiency and insufficiency and their relationship with sun exposure among T2DM patients in Johor, Malaysia.

## Materials and methods

This cross-sectional study conducted from September 10, 2022, to September 5, 2023, included 330 T2DM patients across seven public healthcare facilities in Johor which is a southern region of Peninsular Malaysia. Segamat district was selected because of its high population density in Johor. The majority of respondents were Malay, followed by Chinese and Indian communities, reflecting the ethnic diversity found in Malaysia. The existing data indicate a significant prevalence of T2DM in Segamat and this district has a mix of urban and rural populations.

The inclusion criteria were as follows: (i) patients aged 18 years or older, (ii) diagnosed with type 2 diabetes mellitus for at least six months, (iii) able to understand either Malay or English, and (iv) have attended at least one follow-up visit. The respondents with comorbidities were included. Those who were pregnant were excluded from the study. Data on sun exposure were gathered using a validated questionnaire that assessed the duration of exposure on weekdays and weekends and the body parts exposed to sunlight when outside the house [12]. Sun exposure questionnaire was used in this study. This questionnaire measures the duration each respondent spends outdoors between 10 am and 4 pm. The total exposure time is categorized into ≤30 minutes and >30 minutes. A sun exposure questionnaire was used for 1 hour, >1 hour to 2 hours, and more than 2 hours per day. Additionally, the body parts exposed to sunlight outdoors are recorded according to the following categories: (a) hands and face only, (b) wearing a short-sleeved shirt, (c) wearing a T-shirt and shorts, and (d) wearing shorts only.

Data on demographic and socioeconomic factors, including age, gender, ethnicity, total household income, employment status, education level, and marital status, were collected. Each respondent was instructed to fast for at least 8 hours before blood collection, and a 5 mL blood sample was obtained to measure serum vitamin D levels. Blood specimens were received at the specimen reception counter in each health clinic's laboratory. The received specimens were validated by verifying the identity data on the laboratory request form against the identity data on the blood specimen bottles.

The accepted blood samples were centrifuged in a centrifuge machine at 3000 rpm for 10 minutes. After centrifugation, the serum content was checked to ensure sufficient volume and the absence of red blood cell hemolysis for further testing procedures. The blood samples were then sent to the Universiti Kebangsaan Malaysia (UKM) Medical Molecular Institute (UMBI) for further processing. The samples were stored in a storage box at a temperature of 2-10°C with ice packs during transportation.

Serum vitamin D levels were analyzed at the UKM Medical Molecular Biology Institute (UMBI) in Malaysia using the ultra-performance liquid chromatography (UPLC) method. This method is known for its sensitivity and specificity in determining vitamin D concentrations [13]. The liquid chromatography technique, particularly ultra-high-performance liquid chromatography (UPLC), has become increasingly popular in research due to its ability to separate and analyze 25OHD_2_ and 25OHD_3_ molecules individually, even at low concentrations. This makes UPLC more sensitive and specific for vitamin D determination. The separation of these two metabolites is typically performed using a C18 column packed with small-diameter particles, while detection is carried out through spectral analysis. This UPLC method has been developed and validated in the UMBI laboratory following the guidelines set by the International Council for Harmonisation (ICH) of Technical Requirements for Pharmaceuticals for Human Use and the U.S. Food and Drug Administration (FDA) [13].

Sample size determination and dependent variable

The sample size was calculated using two proportions and the Kish formula (1965). The Kish formula (1965) is a widely used statistical method for estimating sample size in survey-based research. It is particularly useful for studies involving proportions, where the prevalence of a condition or characteristic in a population is the key parameter.

Based on previous research by Hong et al. in 2020, the required sample size was determined to be 296, with a statistical power of 0.80 and an alpha level of 0.05 [14]. Adding 10% to account for potential dropout resulted in a target sample size of 320. This study calculated the highest sample size based on the study objective. The serum vitamin D levels were categorized as follows: <30 nmol/L (deficient), 30-50 nmol/L (insufficient), and >50 nmol/L (sufficient) [13].

Data analysis

Data analysis was performed using IBM SPSS Statistics version 27.0 for Windows (Armonk, NY: IBM Corp.). Descriptive statistics were used to describe the sociodemographic characteristics and clinical characteristics. Factors associated with vitamin D deficiency and insufficiency were analyzed using the chi-square test and multinomial logistic regression analysis. All the statistical analyses were carried out at a 95% confidence interval or p<0.05.

## Results

Sociodemographic and clinical characteristics of the respondents

The sociodemographic and clinical characteristics of the respondents are presented in Table 1. The mean age of the respondents was 62.49±10.64 years, with the majority (216, 65.5%) being over 60 years old. Women accounted for 62.7% (n=207) of the total respondents. Regarding ethnicity, Malays constituted the largest group (54.4%, n=180), followed by Chinese and Indians. In terms of education, 45.5% (n=150) of the respondents had completed primary school, 44.9% (n=148) had completed secondary school, and 9.6% (n=32) had attained university-level education. Nearly half of the respondents were employed, and the vast majority (95.8%, n=316) were married. Clinically, 67.9% (n=224) of the respondents had poor glycemic control, with a mean HbA1c of 6.67±1.92%.

**Table 1:** Sociodemographic and clinical characteristics of the respondents (n=330). The data has been represented as mean±SD and n (%).

Variables		Mean (SD)	Frequency (percentage)
Age group (years)	<60	62.49 (10.64)	114 (35.5)
	≥60		216 (65.5)
Gender	Men	-	123 (37.3)
	Woman		207 (62.7)
Race	Malay	-	180 (54.5)
	Chinese		93 (28.2)
	Indian		57 (17.3)
Education level	Primary education	-	150 (45.5)
	Secondary education		148 (44.9)
	Tertiary education		32 (9.6)
Marital status	Married	-	316 (95.8)
	Unmarried		14 (4.2)
Status of working	Working	-	160 (48.5)
	Not working		170 (51.5)
HbA1c (%)	≤6.5%	7.67 (1.92)	106 (32.1)
	>6.5%		224 (67.9)

Serum vitamin D levels in patients with T2DM

The mean vitamin D levels among respondents was 49.26 nmol/L (SD 15.21). Overall, 58.5% (n=193) of the respondents had low serum vitamin D levels with 12.1% (n=40) of the respondents classified as vitamin D deficient, 46.4% (n=153) as vitamin D insufficient (Table 2).

**Table 2:** Serum vitamin D levels of the respondents (n=330). The data has been represented as mean±SD and number (%).

Serum vitamin D level (nmol/L)	Mean (SD)	Frequency (percentage)
<30 nmol/L (deficient)	49.26 (15.21)	40 (12.1)
30-50 nmol/L (insufficient)		153 (46.4)
>50 nmol/L (sufficient)		137 (41.5)

Sun exposure patterns

During weekdays, 39.7% (n=131) of the respondents reported sunlight exposure ≤30 minutes, while 17.0% (n=57) reported exposure exceeding 2 hours. On weekends, a similar pattern emerged, with 41.5% (n=137) exposed ≤30 minutes and 11.2% (n=37) exposed more than 2 hours. Regarding body surface area exposed to sunlight, 43.9% (n=145) of the respondents exposed their face, arms, and while 28.8% exposed only their face and hands (Table 3).

**Table 3:** Sun exposure pattern among the respondents (n=330). The data has been represented as n (%).

Sunlight exposure (outdoors daily from 10 am to 4 pm)		Frequency (percentage)
During weekdays	≤30 minutes	131 (39.7)
	>30 minutes to 1 hour	97 (29.4)
	>1-2 hours	46 (13.9)
	>2 hours	56 (17.0)
During the weekend	≤30 minutes	137 (41.5)
	>30 minutes to 1 hour	109 (33.1)
	>1-2 hours	47 (14.2)
	>2 hours	37 (11.2)
Parts that are exposed to sunlight when going out of the house	Face and hands	95 (28.8)
	Face, arms, and hands	145 (43.9)
	T-shirt and shorts	90 (27.3)

The bivariate analysis showed a significant association between limited sun exposure (less than 30 minutes during weekends and weekdays) and vitamin D deficiency and insufficiency (Table 4). A multinomial analysis demonstrated that reduced sun exposure during these periods was significantly associated with an increased likelihood of vitamin D deficiency and insufficiency (Table 5). The respondents who were exposed to sunlight for ≤30 minutes during weekdays had higher odds of having vitamin D insufficiency (adjusted odds ratio {AOR}: 2.28, 95% CI: 1.09-4.88, p=0.029) and vitamin D deficiency (AOR: 4.12, 95% CI: 1.02-6.64, p=0.014). Similarly, participants with sun exposure for ≤30 minutes during the weekend had higher odds of vitamin D insufficiency (AOR: 3.82, 95% CI: 1.14-2.85, p=0.035) and vitamin D deficiency (AOR: 4.26, 95% CI: 1.14-2.85, p=0.039).

**Table 4:** Bivariate analysis for factors associated with vitamin D deficiency and insufficiency among T2DM patients. *P-value <0.05 is considered significant. T2DM: type 2 diabetes mellitus The chi-square test was performed to calculate the p-values.

Variables		Normal, n (%)	Vitamin D insufficiency, n (%)	Vitamin D deficiency, n (%)	Chi-square value	p-Value
Age group (years)	<60	45 (35.2)	67 (52.3)	16 (12.5)	3.674	0.159
	≥60	92 (45.5)	86 (42.6)	24 (11.9)		
Gender	Men	51 (41.5)	58 (47.2)	14 (11.4)	0.115	0.886
	Woman	86 (41.5)	95 (45.9)	26 (12.6)		
Race	Malay	84 (46.7)	81 (45.0)	15 (8.3)	3.816	0.355
	Chinese	33 (35.5)	42 (45.2)	18 (19.4)		
	Indian	20 (35.1)	30 (52.6)	7 (12.3)		
Education level	Primary education	60 (40.0)	68 (45.3)	22 (14.7)	3.816	0.432
	Secondary education	67 (45.3)	67 (45.3)	14 (9.5)		
	Tertiary education	10 (31.3)	15 (56.3)	4 (12.5)		
Marital status	Married	128 (40.5)	150 (47.5)	38 (12.0)	3.843	0.146
	Unmarried	9 (64.3)	3 (21.4)	2 (14.3)		
Status of working	Working	59 (36.9)	80 (50.0)	21 (13.1)	2.755	0.252
	Not working	78 (45.9)	73 (42.9)	19 (11.2)		
HbA1c (%)	≤6.5%	42 (39.6)	52 (49.1)	12 (11.3)	0.462	0.794
	>6.5%	95 (42.4)	101 (45.1)	28 (12.5)		
Parts that are exposed to sunlight when going out of the house	Face and hands	45 (47.4)	43 (45.3)	7 (7.4)	4.413	0.353
	Face, arms, and hands	55 (37.9)	71 (49.0)	19 (13.1)		
	T-shirt and shorts	37 (41.1)	39 (43.3)	14 (15.6)		
Sunlight exposure during the weekdays	≤30 minutes	64 (48.9)	33 (25.2)	34(25.9)	9.263	0.046*
	>30 minutes to 1 hour	38 (39.2)	45 (46.4)	14 (14.4)		
	>1-2 hours	17 (37.0)	22 (47.8)	7 (15.2)		
	>2 hours (reference)	18 (32.1)	33 (58.9)	5 (8.9)		
Sunlight exposure weekend	≤30 minutes	65 (47.4)	54 (39.4)	18 (13.1)	8.365	0.031*
	>30 minutes to 1 hour	39 (35.8)	56 (51.4)	14 (12.8)		
	>1-2 hours	17 (36.2)	23 (48.9)	7 (14.9)		
	>2 hours	16 (43.2)	10 (27.0)	11 (29.8)		

**Table 5:** Multinomial logistic regression for factors associated with vitamin D deficiency and vitamin D insufficiency among T2DM patients. *P-value <0.05 is considered significant. CI: confidence interval; T2DM: type 2 diabetes mellitus The multinomial logistic regression was performed to calculate the p-values.

Variables		Vitamin D insufficiency			Vitamin D deficiency		
		Adjusted odd ratio	95% CI	p-Value	Adjusted odd ratio	95% CI	p-Value
Sunlight exposure during the weekdays	≤30 minutes	2.28	1.09-4.88	0.029*	4.12	1.02-6.64	0.014*
	>30 minutes to 1 hour	0.42	0.11-1.76	0.146	0.15	0.11-1.76	0.208
	>1-2 hours	0.32	0.25-1.46	0.387	0.37	0.65-2.46	0.387
	>2 hours	1.00	1.09-4.88	0.029*	1.00	1.14-2.85	-
Sunlight exposure during the weekend	≤30 minutes	3.82	1.14-2.85	0.035*	4.26	0.23-1.90	0.039*
	>30 minutes to 1 hour	0.75	0.73-5.68	0.142	0.26	0.135-1.34	0.449
	>1-2 hours	0.86	0.73-6.86	0.175	0.14	1.14-2.85	0.175
	> 2 hours	1.00	1.14-2.85	-	1.00	-	0.039*

## Discussion

The total number of respondents in this study was 330 patients with type 2 diabetes mellitus (T2DM). This study reveals that 12.1% of respondents had vitamin D deficiency, while 46.4% had vitamin D insufficiency, consistent with previous studies indicating that T2DM patients are at a higher risk of having vitamin D deficiency and insufficiency [8]. This is the first study to measure vitamin D levels in T2DM populations in Malaysia. This relationship between vitamin D deficiency and insulin resistance in T2DM patients has been widely documented [15]. Numerous studies have explored the relationship between vitamin D and pancreatic β-cell function. The β-cells express vitamin D receptors and play an important role in insulin expression [16]. Since calcium-mediated insulin secretion is dependent on vitamin D, low vitamin D status may impair glucose homeostasis [16].

Additionally, this study found that limited sunlight exposure of less than 30 minutes during weekdays and weekends was significantly associated with low serum vitamin D levels. The analysis revealed that sun exposure for less than 30 minutes during weekdays had a 1.5 times greater risk of having low serum vitamin D levels. Similarly, those exposed to sunlight for less than 30 minutes during the weekend had a 4.6 times greater risk of having low serum vitamin D levels. This finding aligns with a study conducted in India, which demonstrated the duration of sunlight exposure significantly impacts vitamin D levels [17]. The longer amount of time spent outside during the weekdays and on the weekends was associated with higher mean vitamin D levels [17]. Many people are engaged in outdoor work-related or daily routine activities on weekdays and this condition contributes to differences in sun exposure [3].

Studies in Saudi Arabia have shown that individuals who receive regular exposure to sunlight have a reduced risk of vitamin D deficiency in contrast to those who were minimally exposed [18]. A clinical trial further demonstrated that individuals who expose 20-30% of their body surface, such as the hands, arms, and legs, for 30-60 minutes per day, three times a week during the summer season, showed an increase in serum vitamin D levels [19]. These findings underscore the critical role of sunlight in the synthesis of vitamin D. Vitamin D can be obtained either through endogenous production in the skin following sunlight exposure or through external sources, such as a balanced diet or supplements [20]. Natural dietary sources of vitamin D are typically insufficient to fulfill the body’s physiological requirements, highlighting the importance of adequate sunlight exposure for maintaining optimal vitamin D levels [21].

Therefore, the primary source of vitamin D is its synthesis in the skin following exposure to sunlight, which is crucial [22]. While sunlight facilitates the skin's synthesis of vitamin D, this process is influenced by several factors, including age, skin pigmentation, clothing style, and religious practices [22,23]. Considering religious aspects, only the face and hands are typically permitted to be exposed to sunlight during daily activities [24]. Wearing long-sleeved clothing, headscarves, or full-body coverings reduces skin exposure to sunlight, limiting vitamin D synthesis. Cultural and religious dress codes may increase deficiency rates in certain populations [2]. Based on our findings, for clothing, 44% of the respondents chose the face, arms, and hands for the parts exposed to sunlight when leaving the house, followed by the face and hands by 29%. However, our study revealed that the exposed area was not significantly associated with serum vitamin D levels in T2DM patients.

Despite sunlight exposure being critical for vitamin D synthesis, it is also essential for healthy bones, the immune system, and mental well-being. Reduced sunlight exposure, often a consequence of sedentary lifestyles or urban living, has been linked to an elevated risk of multiple health problems [25]. According to the literature, the majority of individuals with T2DM engage in moderate levels of physical activity [26]. This is consistent with a nationwide study, which showed that among T2DM individuals, 40% do not regularly participate in adequate physical activity [27]. Reduced physical activity indirectly leads to lower exposure to sunlight, which has significant health implications [28]. Sun exposure requires determining the optimum timing of the day, season, latitude, weather, and skin color to achieve the necessary vitamin D levels [4,21].

In Malaysia, due to cultural norms that favor lighter skin tones and worry about the risk of skin cancer, people frequently spend less time outside and steer clear of prolonged sun exposure. This tendency results in more individuals choosing to stay indoors [29]. Many individuals take measures to minimize sun exposure due to prevailing cultural preferences that prioritize maintaining a lighter complexion, mainly to prevent facial skin from darkening [1,2]. A study of South Asian adults found that respondents with darker skin tones required longer sun exposure which was approximately 51 minutes on average for a day [30]. Further longitudinal studies with data on the intensity of sunlight exposure, lifestyle, clothing preferences, and skin type are needed for a more thorough investigation into the appropriate and exact duration of sunlight exposure for the population, particularly for T2DM patients.

Other factors must be considered in the synthesis of vitamin D including skin pigmentation. As vitamin D is synthesized in the skin following sunlight exposure and is highly correlated with skin color, the Malay and Indian populations in Malaysia are at higher risk of lower vitamin D status [1]. Melanin acts as a natural sunscreen, reducing the skin’s ability to produce vitamin D [2]. Individuals with darker skin require longer sun exposure to synthesize adequate vitamin D than those with lighter skin. Furthermore, aging reduces the skin's ability to synthesize vitamin D due to a decline in 7-dehydrocholesterol levels, the precursor needed for conversion under UVB exposure [2,4]. Older adults often have limited sun exposure due to mobility issues and lifestyle factors, further increasing the risk of deficiency [2,4].

In terms of lifestyle, many T2DM patients engage in moderate or low physical activity levels, with a significant proportion preferring indoor activities due to health concerns, environmental factors, or cultural preferences [2,5,21]. Therefore, lesser physical activity limits sun exposure and contributes to poor glucose control and increased insulin resistance. Since vitamin D plays a role in insulin sensitivity and β-cell function, inadequate sun exposure and sedentary behavior may further exacerbate metabolic dysfunction in T2DM patients. Occupational commitments, clothing styles, and sedentary lifestyle concerns also influence these patterns, making it essential to develop tailored strategies to promote both physical activity and optimal vitamin D levels among at-risk populations.

Strength and limitation

This is the first study in Malaysia to examine vitamin D levels in T2DM populations and the relationship between sun exposure and vitamin D status among T2DM patients. This study's use of ultra-performance liquid chromatography (UPLC) for vitamin D analysis enhances the accuracy and reliability of the findings. The application of multinomial logistic regression allows for the adjustment of confounders, strengthening the validity of the associations observed; however, certain limitations should be acknowledged. This cross-sectional study prevents the establishment of the causal relationship between sun exposure and vitamin D levels. Additionally, a self-reported questionnaire will induce potential bias, which affects the accuracy of the reported exposure duration. Furthermore, as the study was conducted exclusively in Johor, the findings may not be generalizable to other Malaysian populations with different cultural or environmental factors influencing sun exposure habits.

## Conclusions

Vitamin D deficiency and insufficiency are prevalent among T2DM patients in Johor, Malaysia with limited sun exposure identified as a key modifiable risk factor. Given the established link between vitamin D deficiency and insulin resistance, targeted public health interventions are urgently needed. Strategies should include public awareness campaigns to promote optimal sun exposure, particularly among individuals at risk of deficiency. Further research should explore the longitudinal effects of vitamin D deficiency on metabolic outcomes in T2DM patients and evaluate effective interventions to improve vitamin D status in this population. To enhance public awareness and promote adequate vitamin D levels, public health campaigns could include the following components, such as community education programs. These could involve organizing workshops and health talks in clinics, community centers, and workplaces to educate people on the importance of vitamin D and safe sun exposure practices. Distributing educational materials (brochures, posters, infographics) in local languages would help improve accessibility. Additionally, media and digital outreach would play a key role. Social media campaigns featuring short videos, expert interviews, and infographics could help dispel myths about sun exposure and vitamin D. Collaborating with influencers, healthcare professionals, and community leaders would further amplify these messages.
